# Ultrasound-assisted extraction of flavonoids from *Artemisia argyi* folium using deep eutectic solvents: optimization, mechanistic characterization, bioactivity evaluation, and strawberry preservation applications

**DOI:** 10.1016/j.ultsonch.2026.107952

**Published:** 2026-07-07

**Authors:** Nina Bao, Xinyuan Zhao, Lele Li, Marwan M.A. Rashed, Weifeng Yuan

**Affiliations:** a School of Biological and Food Engineering, Suzhou University, Suzhou 234000, China; b Collaborative Technology Service Center for the High-Value Processing of Green Agricultural Products (Prepared Foods) in the Yangtze River Delta Region, Suzhou University, Suzhou 234000, China; c College of Food Science and Technology, Shanghai Ocean University, Shanghai 201306, China

**Keywords:** NADESs-UAE, Flavonoids, *Artemisia argyi* folium, Antioxidant activity, α-Glucosidase/α-amylase inhibition, Strawberry preservation

## Abstract

This study presents an efficient and environmentally sustainable technique for extracting flavonoids from *Artemisia argyi* folium, employing natural deep eutectic solvents combined with ultrasound-assisted extraction (NADESs-UAE). Among the ten DES systems evaluated, the Choline Chloride-Lactic Acid mixture (ChCl-LA, molar ratio 1:1, designated as DES-3) yielded the highest extraction of 93.39  mg of rutin equivalents (RE) per gram of dry weight (DW). This was much higher than the yields obtained with traditional 60% ethanol or water extraction methods. Response surface methodology (RSM) was used to optimize key extraction variables, including water content, extraction time, temperature, and liquid-to-solid ratio. Optimal conditions included 51.5  g/100  g water content, 56  min extraction time, and an extraction temperature of 59 °C. The liquid-to-solid ratio reached 48  mL/g under optimized conditions. Under these conditions, the predicted flavonoid yield was 99.27  mg RE/g DW, whereas the experimental value was 98.71 ± 0.66  mg RE/g DW, confirming the model’s reliability. Kinetic investigations demonstrated that the extraction process followed a pseudo-second-order kinetic model, indicating enhanced diffusion-assisted mass transfer under ultrasonic cavitation conditions. Scanning electron microscopy (SEM) analysis revealed that DES-3 induced pronounced cellular disruption, thereby facilitating enhanced flavonoid extraction. Fourier-transform infrared (FT-IR) spectroscopy further confirmed the presence of strong hydrogen-bonding interactions between ChCl-LA and flavonoid compounds. Compared with conventional solvent systems, the ChCl-LA extract exhibited superior antioxidant activity, including DPPH radical-scavenging activity (959.98 ± 9.59  mg Trolox/g DW), as well as enhanced α-glucosidase inhibition (91.8% at 125  μg/mL) and antibacterial activity. Furthermore, treatment with AAF extracts effectively improved strawberry firmness and enhanced POD activity during storage, thereby prolonging strawberry shelf life. Overall, the findings demonstrate that NADESs-UAE represents a promising green extraction strategy for the efficient recovery and practical utilization of flavonoids from Artemisia argyi folium.

## 1 Introduction

*Artemisia argyi* is a perennial herb belonging to the *Asteraceae* family and is widely distributed across temperate regions of Europe, Asia, and North America [37]. Its dried leaves, known as *Artemisia argyi* Folium (AAF), have been extensively used in traditional Chinese medicine due to their broad pharmacological activities, including hypoglycemic, anti-inflammatory, antitumor, antioxidant, antibacterial, and antifatigue effects [23], [56], [60], [64], [72], [13], [14], [15]. These bioactivities are mainly attributed to its rich phytochemicals, particularly flavonoids, which represent one of the most important functional constituents of AAF. Previous studies have confirmed that AAF-derived flavonoids exhibit strong antioxidative, anti-inflammatory, and anticancer activities, highlighting their potential applications in functional foods and pharmaceuticals [5], [57]. As the demand for natural bioactive compounds continues to increase, the development of efficient, environmentally friendly, and economically sustainable extraction technologies has become increasingly important for maximizing the utilization of AAF resources.

With the increasing demand for natural bioactive compounds, the development of efficient, sustainable, and environmentally friendly extraction techniques has become essential. Conventional extraction methods such as maceration and Soxhlet extractionare still widely applied. However, these approaches generally require large volumes of organic solvents, long extraction times, and high energy consumption, which often leads to low efficiency and potential degradation of heat-sensitive compounds [20], [28], [35]. In addition, the extensive use of volatile organic solvents raises concerns regarding environmental impact, solvent toxicity, and operational safety. These limitations highlight the need for greener and more efficient extraction strategies.

Nnatural deep eutectic solvents (NADES) have emerged as a promising class of green solvents for natural product extraction. They are typically composed of natural metabolites such as organic acids, sugars, amino acids, and polyols, which act as hydrogen bond donors and acceptors to form eutectic mixtures with melting points lower than those of individual components [20], [22], [41]. Due to their tunable composition, NADES exhibit adjustable physicochemical properties, including polarity, viscosity, and solvation ability, making them suitable for selective extraction of bioactive compounds. In addition, NADES are generally biodegradable, low in toxicity, and capable of strong interactions with phenolic compounds, which enhances their extraction performance compared to conventional organic solvents. However, their relatively high viscosity can limit solvent diffusion into plant matrices, resulting in reduced mass transfer efficiency. Therefore, process intensification is required to overcome these limitations [20], [22], [28].

Ultrasonic-assisted extraction (UAE) has been widely applied as an effective intensification technique due to its high efficiency, short extraction time, and low energy consumption [20], [24]. The primary mechanism of UAE is acoustic cavitation, which generates microjets and shock waves that disrupt plant cell walls, enhance membrane permeability, and improve solvent penetration. These effects significantly reduce mass-transfer resistance and accelerate the release of intracellular compounds under relatively mild conditions, thereby minimizing thermal degradation of bioactive constituents [4], [45], [47]. Compared with microwave-assisted extraction, UAE provides more uniform energy distribution and better compatibility with viscous solvents such as NADES. Accordingly, the combination of NADES and UAE represents a promising green extraction strategy for flavonoid recovery. Consequently, NADESs-UAE represents a promising strategy for improving flavonoid extraction efficiency and sustainability in future industrial applications [2], [4]. Because flavonoid extraction efficiency is influenced by multiple interacting variables, including water content, extraction time, extraction temperature, and liquid-to-solid ratio, statistical optimization is necessary to accurately determine the optimal extraction conditions. Response Surface Methodology (RSM) is widely employed for extraction optimization because it enables simultaneous evaluation of independent variables and their interactions while minimizing the number of experimental trials [46]. Compared with conventional one-factor-at-a-time approaches, RSM provides more reliable mathematical modeling, predictive capability, and more efficient process optimization for complex extraction systems, such as NADES-UAE extraction.

Despite notable progress in extraction techniques, the underlying mechanisms driving ultrasound-enhanced extraction within NADES systems have received limited scholarly attention. Specifically, the impact of ultrasonic cavitation on mass transfer dynamics, diffusion properties, and extraction kinetics has not been thoroughly examined in a systematic manner. Understanding extraction kinetics is vital, as it offers critical insights into diffusion mechanisms, rate-limiting steps, and process intensification, all of which are essential for reactor design, mathematical modeling, and the scaling up of industrial processes. Moreover, although methods such as scanning electron microscopy (SEM) and Fourier-transform infrared spectroscopy (FT-IR) have been sporadically employed to assess structural alterations during extraction, these techniques have seldom been combined with kinetic analyses to clarify how ultrasonic cavitation influences plant microstructure and facilitates the release of intracellular flavonoids [21], [31], [50]. Consequently, there remains a significant gap in the literature regarding a comprehensive study that integrates extraction optimization, kinetic modeling, structural characterization, and mechanistic elucidation for the extraction of flavonoids from *Artemisia argyi* Folium.

In addition to their antioxidant and enzyme-inhibitory activities, flavonoid-rich extracts from *Artemisia argyi* folium may also exhibit considerable potential as natural food preservatives due to their antioxidant and antimicrobial properties. Strawberry fruits are highly susceptible to microbial spoilage, oxidative deterioration, and rapid softening during storage. Therefore, evaluating the preservation potential of AAF extracts in strawberries may provide additional insight into their practical applications as natural bioactive preservatives in food systems.

Based on these considerations, the present study aims to develop an efficient and sustainable extraction strategy for flavonoids from Artemisia argyi Folium using NADES combined with ultrasound-assisted extraction. Different NADES formulations were screened to identify the most effective solvent system, followed by optimization of extraction parameters using Response Surface Methodology (RSM). Extraction kinetics were then analyzed to elucidate mass-transfer behavior and mechanism. In addition, scanning electron microscopy (SEM) and Fourier-transform infrared spectroscopy (FT-IR) were employed to investigate structural changes in plant tissues and to clarify the synergistic effects of NADES and ultrasound. Finally, the antioxidant, antibacterial, enzyme-inhibitory, and strawberry preservation activities of the obtained extracts were evaluated to establish their functional potential in food applications. This work provides mechanistic insights into ultrasound-assisted NADES extraction and offers a green and efficient strategy for the valorization of *Artemisia argyi* flavonoids.

## 2 Materials and methods

### 2.1 Plant materials and chemical reagents

#### 2.1.1 Plant materials

*Artemisia argyi* was obtained from a locally cultivated farm in Suzhou, Anhui Province, China. The leaves were handpicked, washed with ultrapure water, and air-dried at 40 °C for 48 h to constant weight. They were subsequently ground into a fine powder with a Chinese herbal medicine pulverizer (XL-04B, Guangzhou, China) and passed through 40-mesh sieves. The powdered sample was preserved at –20 °C before analysis.

Strawberries (*Fragaria × ananassa* ‘Red Face’) were handpicked at the red ripening stage from a local farm in Suzhou, Anhui Province, China, and immediately transported to the laboratory. Fruits with uniform color and size, free from damage or disease, were selected as experimental samples and then cooled in air at 0℃ for 2 hours to remove field heat.

#### 2.1.2 Chemical reagents

Rutin, sodium nitrite, aluminum nitrate, H_2_O_2_ and guaiacol were obtained from Shanghai Aladdin Biotechnology Co., Ltd. Ethanol was obtained from Anhui Ante Food Co., Ltd. Sodium hydroxide, sodium chloride, hydrochloric acid, potassium chloride, ethylene glycol, potassium dihydrogen phosphate, disodium hydrogen phosphate, glycine, ammonium acetate, sodium acetate, and glucose were sourced from Sinopharm Chemical Reagent Co., Ltd. All other analytical-grade chemicals utilized in this investigation were purchased from Shanghai Macklin Chemistry Co., Ltd.

### 2.2 Preparation of NADESs

Ten different NADESs were formulated by combining various HBAs and HBDs via heating and stirring, as previously described [20]. Two components were weighed at the appropriate molar ratio, mixed, and stirred while being heated at 80 °C in sealed flasks with a magnetic stirring bar until stable, transparent, and colorless liquids formed. The selected molar ratios were based on preliminary stability and homogeneity evaluations, as well as previous literature reports, to obtain transparent NADES systems with suitable viscosity and extraction performance. A specified quantity of water was then added to the NADESs to facilitate handling by reducing their viscosity. The prepared NADESs were coded as shown in Table 1.

**Table 1:** Composition of NADESs.

No.	Abbreviation	Composition HBA: HBD	Molar ratio
DES-1	ChCl-Gly	Choline Chloride: Glycerol	1:2
DES-2	LA-SA	Lactic Acid: Sodium Acetate	3:1
DES-3	ChCl-LA	Choline Chloride: Lactic Acid	1:1
DES-4	ChCl-EG	Choline Chloride: Ethylene Glycol	1:2
DES-5	ChCl-PG	Choline Chloride: Propylene Glycol	1:2
DES-6	LA-Glu	Lactic Acid: Glucose	5:1
DES-7	TA-EG	Tartaric Acid: Ethylene Glycol	1:1
DES-8	MA-Gly	Malic Acid: Glycerol	1:1
DES-9	LA-AA	Lactic Acid: Ammonium Acetate	3:1
DES-10	LA- Gly	Lactic Acid: Glycine	4:1

### 2.3 NADESs-UAE Process

#### 2.3.1 Selection of NADESs

NADESs are considered effective for extraction due to their adjustable viscosity, high biodegradability, and strong extraction capability. Therefore, various types of NADESs, such as ChCl-Gly, LA-SA, ChCl-LA, ChCl-EG, ChCl-PG, LA-Glu, TA-EG, MA-Gly, LA-AA, and LA- Gly were tested in a preliminary experiment to select the optimal type. Approximately 1.0 g of AAF was mixed with 40 mL of NADES (70% v/v), and the mixture was ultrasonicated in an ultrasonic bath (KS-500XDS, Kunshan Jielimei Ultrasonic Instrument Co. Ltd., Jiangsu, China) at 50 °C for 45 min (up to 500 W, 40 kHz).

#### 2.3.2 Total flavonoid content (TFC)

The aluminum chloride colorimetric assay was employed for the determination of TFC [59], with minor modifications. In brief, 500 µL of water-diluted AAF extracts was mixed with 30 µL of 5% NaNO_2_ solution in a 2 mL tube, followed by incubation at ambient temperature for 5 min. Then, 30 µL of 10% AlCl_3_ solution was added, followed by an additional 6 min of incubation. Subsequently, 200 µL of 1 M NaOH was added, and distilled water was added to bring the final volume to 1 mL. The mixture was vortexed thoroughly and incubated at 30 °C for 30 min. Absorbance was recorded at 510 nm. A calibration curve was established with rutin at 10–100 μg/mL. (*R^2^* = 0.9998). Data were expressed as milligrams of rutin equivalents (RE) per gram of dry weight (mg RE/g DW).

#### 2.3.3 Experimental design

Based on the most efficient NADES selected in Section 2.3.1, single-factor experiments were carried out to optimize the extraction conditions, including water content, extraction time, extraction temperature, and liquid-to-solid ratio, for maximizing TFC yield. Preliminary screening experiments showed that pH and ultrasonic power exerted relatively minor effects on flavonoid extraction under the selected conditions; therefore, these parameters were fixed at pH 8 and 400 W, respectively, during subsequent experiments. According to the preliminary experiments, four factors significantly influenced TFC yield: water content (10–90%), extraction time (10–110 min), extraction temperature (30–80 °C), and liquid-to-solid ratio (10–50 mL/g). RSM (CCD) was employed to evaluate the interactive effects of these factors. A total of 30 experiments, including 6 replicates at the central point, were carried out as shown in Table 2.

**Table 2:** RSM: design and results.

Run	A: Water content (g/100 g)	B: Extraction time (min)	C: Extraction temperature (°C)	D: Liquid-to-solid ratio (mL/g)	Expermintal values of TFC (mg RE/g)	Predicted values of TFC (mg RE/g)
1	70	70	70	50	54.58	54.57
2	60	50	60	40	94.85	94.36
3	80	50	60	40	58.32	57.97
4	60	50	60	40	94.83	94.36
5	40	50	60	40	85.59	86.04
6	70	30	50	30	84.96	84.73
7	70	70	50	50	76.05	76.78
8	50	70	70	50	89.48	89.98
9	50	30	70	50	80.85	80.77
10	60	50	60	40	94.43	94.36
11	60	90	60	40	68.51	67.77
12	70	70	70	30	43.39	44.05
13	70	30	70	30	49.49	49.47
14	70	30	70	50	54.39	54.09
15	60	50	60	20	73.72	73.95
16	50	70	50	30	80.95	81.52
17	60	50	60	40	93.69	94.36
18	50	70	70	30	68.29	67.86
19	60	10	60	40	62.31	63.15
20	60	50	60	40	93.83	94.36
21	70	30	50	50	75.41	75.47
22	50	70	50	50	90.11	89.77
23	60	50	60	40	94.52	94.36
24	50	30	70	30	65.01	64.55
25	60	50	40	40	87.31	87.39
26	60	50	80	40	52.32	52.34
27	50	30	50	50	80.12	79.73
28	60	50	60	60	86.95	86.82
29	70	70	50	30	80.41	80.13
30	50	30	50	30	77.75	77.39

Experimental design was performed using Design-Expert software (version 13), regression analysis, and graphical representation. Analysis of variance (ANOVA) was applied to test for notable differences among linear, quadratic, and interaction terms, and to assess the statistical significance of the fitted model and regression coefficients.

#### 2.3.4 Model validation

For model validation, the experimental value obtained under the optimal conditions, as selected by Design-Expert software (version 13), was compared with the model’s predicted value.

### 2.4 Extraction kinetics

Kinetic studies of the extraction process have predominantly demonstrated that it follows a second-order kinetic model [20], [49]. The extraction kinetics were investigated under optimized NADES-UAE conditions: 51.5 g/100 g water content, 56 min extraction time, 59 °C extraction temperature, and a liquid-to-solid ratio of 48 mL/g. Ultrasonic power was maintained at 400 W, and the pH was kept at 8 throughout the extraction process. Samples were collected at predetermined time intervals, and the TFC index was subsequently analyzed. According to prior research [20], the dissociation rate of total flavonoids from AFFs powder can be described by the following second-order kinetic equation:

(1) $${\text{dC}}_{\text{t}}/\text{dt}\;=\;\text{k}{\left({\text{C}}_{\text{s}}-{\;\text{C}}_{\text{t}}\right)}^{2}$$

where, C_t_ denotes the concentration of total flavonoid content (TFC, mg RE/g DW) at a predetermined time t (min), C_s_ represents the saturated concentration of TFC, and k is the second-order extraction rate constant (g·min^−1^·mg^−1^). Equation (1) is typically integrated over the boundary conditions (t = 0 to t and Ct = 0 to Ct), and then linearized to yield the following expression:

(2) $$\text{t}/{\text{C}}_{\text{t}}=1/\text{k}\cdot {\text{C}}_{\text{s}}^{2}+\;\text{t}/{\text{C}}_{\text{s}}=1/\text{h}\;+\;\text{t}/{\text{C}}_{\text{s}}$$

where, h denotes the initial extraction rate (mg·min^−1^·mg^−1^) as t and C_t_ approach zero. Consequently, the parameters C_s_, h, and k can be determined from the slope and intercept of the linear regression of t/C_t_ plotted against t.

### 2.5 FT-IR analysis

FT-IR spectroscopy (Nicolet iS50 Spectrometer, Thermo Scientific, Germany) was used to analyze functional groups of AAF extracts. Lyophilized extracts were incorporated into KBr discs, and spectral data were acquired in the range of 4000–400 cm^−1^.

### 2.6 SEM analysis

Samples were washed three times in alternating cycles of ethanol and water, lyophilized (Model LGJ-12 N, Yaxingyike, China), mounted on aluminum stubs, sputter-coated with a gold layer, and examined using a scanning electron microscope (SEM; SU1510, Hitachi, Japan).

### 2.7 *In vitro* antioxidant assays

Antioxidant activities, including DPPH, ABTS, ferric reducing antioxidant power (FRAP), and reducing activity (RA), were assayed with minor modifications following the methods of Bao et al.[8]and Fu et al.[20]. Values were quantified against a Trolox calibration curve and are presented as milligrams of Trolox equivalents per gram of AAF dry weight (mg TE/g DW).

### 2.8 *In vitro* enzyme inhibitory activities

The α-glucosidase and α-amylase inhibitory activities were assayed following the protocol previously outlined by Zhang and Bao [7], [70]with minor adjustments. For α-glucosidase inhibition, absorbance was measured at 405 nm; for α-amylase inhibition, at 540 nm, using a microplate reader (Thermo Fisher Scientific, model 1510, Shanghai, China).

Inhibitory activity was quantified as the reduction in absorbance relative to the control. To validate the assay, acarbose was employed as a positive control at 10–200 μg/mL.

### 2.9 *In vitro* antibacterial activities

Three foodborne bacteria were used in the bioassays: Gram-positive bacteria (*Staphylococcus aureus* and *Bacillus subtilis*) and a Gram-negative bacterium (*Escherichia coli*), selected as target microbes to evaluate the antibacterial activities of the AAF extracts. The evaluation was performed using the Oxford cup method as previously described by Li [33]. Bacterial suspensions with a concentration of 1.0 × 10^7^ CFU/mL were inoculated into soft agar media. Once the soft agar had solidified, Oxford cups filled with AAF extract solutions and a negative control (Choline Chloride-Lactic Acid) were placed onto the agar plates. The inhibition zone diameters were determined after 24 h of incubation at 37 °C.

### 2.10 Application of AAF extracts in the preservation of strawberries

2.10.1 Treatment of Strawberries

A total of 150 strawberries were selected and randomly assigned to five groups (I, II, III, IV, and CK). The AAF extracts, obtained under optimized extraction conditions, were diluted at ratios of 1:10, 1:5, 1:2.5, and 1:1.25 to prepare preservation solutions corresponding to groups I through IV, respectively. Distilled water was used for the control group (CK). Strawberries in each treatment group were immersed in their respective solution for 10 min, air-dried at 20 °C for 30 min, and subsequently stored on plastic trays at 20 °C for 7 days to assess shelf-life parameters.

#### 2.10.1 Measurement of strawberry firmness

Firmness of the strawberry samples was determined by a texture analyzer (TMS-PRO, Food Technology Corporation, Virginia, USA). From each group, ten strawberries were randomly selected, and firmness was measured around the equator of each fruit using a P/5 cylindrical probe. The probe operated at 1 mm/s to a penetration depth of 5 mm. Firmness was quantified as the maximum force recorded, expressed in Newtons (N). The experiment included three biological replicates and three technical replicates.

#### 2.10.2 Peroxidase (POD) enzyme activity assay

Peroxidase activity was evaluated following the protocol outlined by Li et al [34]. The assay mixture consisted of 3 mL of 25 mmol L^−1^ guaiacol, 200 μL of 0.5 mol L^−1^ H_2_O_2_, and 500 μL of POD enzyme extract. Enzyme activity was quantified by monitoring the absorbance variation at 470 nm.

### 2.11 Statistical analyses

The data processing for this research was conducted using GraphPad Prism 8 (GraphPad Software, Inc., San Diego, CA, USA) and Design-Expert 13 (Stat-Ease, Inc., Minneapolis, MN, USA). Kinetic plots were processed using Microsoft Excel 2016. Significant differences (*p* ≤ 0.05) between means were evaluated by one-way ANOVA followed by Duncan’s multiple range test using IBM SPSS Statistics 20. All determinations were carried out in triplicate, with results presented as mean ± standard deviation (SD).

## 3 Results and discussion

### 3.1 Selection of NADESs

**Fig. 1:**
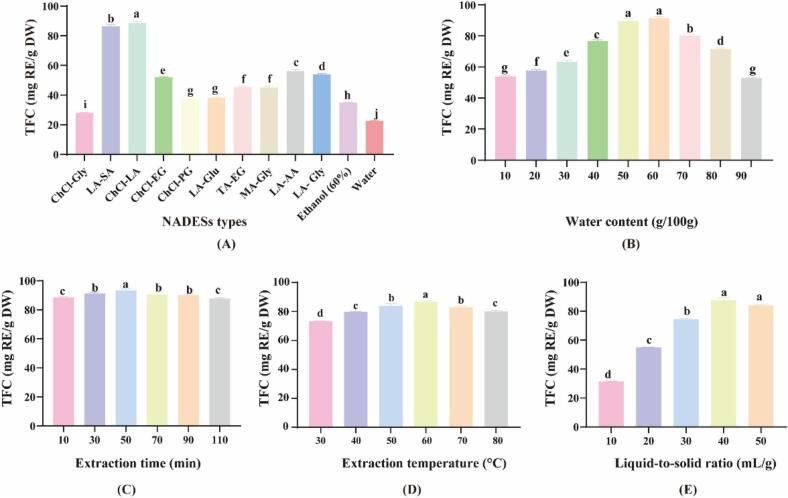
Effect of NADESs and conventional solvents on TFC from AAF (A); effect of water content (B), extraction time (C), extraction temperature (D), and liquid-to-solid ratio (E) on TFC from AAF. Data are shown as means ± SD (n = 6). Different lowercase letters (a, b, c, …, j) above the bars denote significant differences at *p* < 0.05.

The selected NADESs for extracting flavonoid were designed based on their distinct physicochemical properties, including polarity, viscosity, pH, and hydrogen-bonding affinity. Consequently, eighteen NADES formulations were initially prepared. However, only ten remained homogeneous and transparent after storage for 24 h at room temperature (Section 2.2 Table 1), indicating their suitability for further extraction studies. To identify the most effective solvent system, these ten NADESs were evaluated for flavonoid extraction from AAF, using total flavonoid content (TFC) as the response indicator. For comparison, water and 60% ethanol were used as conventional green solvents. As shown in Fig. 1(A), all NADESs exhibited higher extraction efficiency than water, confirming their enhanced solubilization capability. However, only ChCl–Gly performed slightly lower than 60% ethanol. Among all systems, ChCl–lactic acid (ChCl–LA) exhibited the highest TFC yield, achieving approximately 4-fold and 2-fold higher values compared to water and ethanol, respectively.Therefore, ChCl–LA was selected for subsequent experiments. The superior performance of ChCl–LA can be attributed to its strong hydrogen-bonding network, optimal polarity matching with flavonoids, and balanced viscosity, which collectively enhance solute–solvent interactions. In contrast, NADESs with higher viscosity or weaker hydrogen-bonding ability exhibited reduced mass transfer efficiency [20]. Notably, similar enhancement trends have been reported for NADES-based extraction systems in polyphenol recovery [42], confirming that extraction efficiency is strongly dependent on HBD/HBA interactions and solvent microstructure. Furthermore, solvent viscosity is a key factor affecting mass transfer and cavitation during extraction or ultrasonication, which is critical for extraction efficiency [42], [48]. In addition, the molar ratio between hydrogen-bond donors and hydrogen-bond acceptors strongly influences the physicochemical properties of NADES systems, particularly viscosity, polarity, and hydrogen-bonding interactions. Therefore, the molar ratios employed in this study were selected according to preliminary stability assessments and previously reported effective formulations.

These findings clearly demonstrate that ChCl–LA-based NADES provides a highly efficient and environmentally friendly extraction medium for flavonoid recovery from AAF.

### 3.2 Single factors and interactive effects on TFC using NADESs-UAE

#### 3.2.1 Single-factors effects

Based on Section 3.1, ChCl–LA was selected as the optimal NADES for further optimization. The effects of water content, extraction time, temperature, and liquid-to-solid ratio were systematically investigated.

*Effects of water content*. Water content plays a crucial role in tuning NADES physicochemical properties, particularly viscosity and hydrogen-bonding structure. As shown in Fig. 1(B), TFC initially increased and then decreased with increasing water content (10–90 g/100 g), reaching a maximum at 60 g/100 g (other parameters: 40 mL/g, 50 °C, 45 min). This trend indicates that moderate water addition reduces viscosity and enhances mass transfer, thereby facilitating flavonoid diffusion. However, excessive water disrupts the hydrogen-bonding network of NADES, weakening solute–solvent interactions and reducing extraction efficiency.

These findings are consistent with previous studies reporting that NADES performance is strongly governed by the balance between viscosity reduction and structural integrity [48]. Moreover, similar behavior has been observed in DES-based extraction systems, where optimal water content depends on system composition and target solutes [42]. In this study, the optimal water content (60%) suggests a system-specific balance between solvation strength and mass-transfer efficiency.

*Effect of extraction time*. As shown in Fig. 1(C), TFC increased significantly from 10 to 50 min, reaching a maximum of 93.39 mg RE/g, followed by a slight decline at longer extraction times (10–110 min). This behavior reflects a typical rapid extraction phase followed by equilibrium and degradation stages. Initially, ultrasonic cavitation enhances cell disruption, facilitating rapid release of intracellular flavonoids. However, prolonged sonication may induce thermal and oxidative degradation of sensitive phenolic compounds, leading to reduced yield. Similar trends have been reported for ultrasound-assisted extraction of phenolics, where excessive sonication reduces compound stability [27]. Comparable outcomes were found for phenolic compounds extracted from peels of *Carya cathayensis* Sarg using ChCl-MA DES [20].

*Effect of extraction temperature*. Temperature significantly influenced extraction efficiency, as shown in Fig. 1(D). TFC increased from 30 to 60 °C, reaching a maximum of 94.29 mg RE/g, but decreased at higher temperatures [48]. This trend is attributed to two competing effects: enhanced mass transfer due to reduced viscosity and surface tension, and thermal degradation of flavonoids at elevated temperatures. Therefore, 60 °C represents an optimal balance between extraction kinetics and compound stability [12].

*Effect of liquid-to-solid ratio*. As shown in Fig. 1(E), increasing the liquid-to-solid ratio from 10 to 40 mL/g significantly improved TFC, while further increases showed no significant enhancement. This indicates that increasing solvent volume enhances the concentration gradient and solute diffusion driving force, thereby improving extraction efficiency. However, beyond a threshold, the system reaches a mass-transfer saturation state, where additional solvent does not improve intracellular solute release. In addition, excessive solvent volumes may lead to reduced effective collision frequency between solvent and matrix, non-specific adsorption losses, and reduced ultrasonic energy density within the system. These factors collectively explain the plateau effect observed at higher liquid-to-solid ratios [10], [11], [67].

#### 3.2.2 RSM modeling and interaction effects

A central composite design (CCD) was employed to evaluate the interactive effects of four independent variables under conditions derived from preliminary experiments. The experimental TFC values obtained using ChCl–LA are summarized in Table 2. The response variable (TFC) was fitted to the following quadratic polynomial model (Eq. (3):

(3) $${\text{Y}}_{\text{TFC}}=94.36-7.02\;\text{A}+1.15\;\text{B}-8.76\;\text{C}+3.22\;\text{D}-2.18\;\text{AB}-5.60\;\text{AC}-2.90\;\text{AD}-0.20\;\text{BC}+1.48\;\text{BD}+3.47\;\text{CD}-5.59{\;\text{A}}^{2}-7.22{\;\text{B}}^{2}-6.12{\;\text{C}}^{2}-3.49{\;\text{D}}^{2}$$

where A–D represent water content, extraction time, extraction temperature, and liquid-to-solid ratio, respectively.

*Model adequacy and statistical significance*. As shown in Table 3, the regression model exhibited strong statistical significance (*p* < 0.0001), indicating that the model reliably describes the experimental data. The lack-of-fit term was not significant (*p* = 0.4167), confirming model adequacy. In addition, the coefficient of determination (*R^2^* = 0.9966) and the adjusted *R^2^* (0.9934) indicate excellent agreement between experimental and predicted values, with minimal deviation ( < 0.01), demonstrating high predictive accuracy.

**Table 3:** Analysis of Variance (ANOVA), factors, and effects of their interaction.

Term	Sum of Squares	Df	Mean Square	F-value	*p*-value
Model	6073.95	14	433.85	313.55	<0.0001

***Linear***					
A- water content	856.85	1	856.85	619.25	<0.0001
B- extraction time	124.61	1	124.61	90.06	<0.0001
C- extraction temperature	2114.53	1	2114.53	1528.18	<0.0001
D- liquid-to-solid ratio	295.46	1	295.46	213.53	<0.0001

***Interactions***					
AB	0.24	1	0.24	0.17	0.6861
AC	880.32	1	880.32	636.21	<0.0001
AD	87.41	1	87.41	63.17	<0.0001
BC	16.56	1	16.56	11.97	0.0035
BD	0.72	1	0.72	0.52	0.4832
CD	346.91	1	346.91	250.71	<0.0001

***Quadratic***					
A^2^	346.91	1	346.91	250.71	<0.0001
B^2^	637.14	1	637.14	460.46	<0.0001
C^2^	976.67	1	976.67	705.84	<0.0001
D^2^	183.45	1	183.45	132.58	<0.0001
Residual	20.76	15	1.38		
Lack of Fit	14.91	10	1.49	1.27	0.4167
Pure Error	5.85	5	1.17		
Cor Total	6094.71	29			
Std. Dev.	1.18				
R^2^	0.9966				
Adjusted R^2^	0.9934				
Adeq Precision	53.965				
Mean	77.98				
C.V. %	1.51				

*Interaction effects*. Analysis of regression coefficients revealed that all linear (A, B, C, D) and quadratic terms (A^2^, B^2^, C^2^, D^2^), as well as selected interaction terms (AC, AD, BC, CD), were statistically significant (*p* < 0.05). In contrast, interaction terms AB and BD were not significant (*p* > 0.05). These results suggest that water content interacts more strongly with temperature and liquid-to-solid ratio than with extraction time, highlighting the dominant role of solvent physicochemical properties in NADES-UAE systems.

**Fig. 2:**
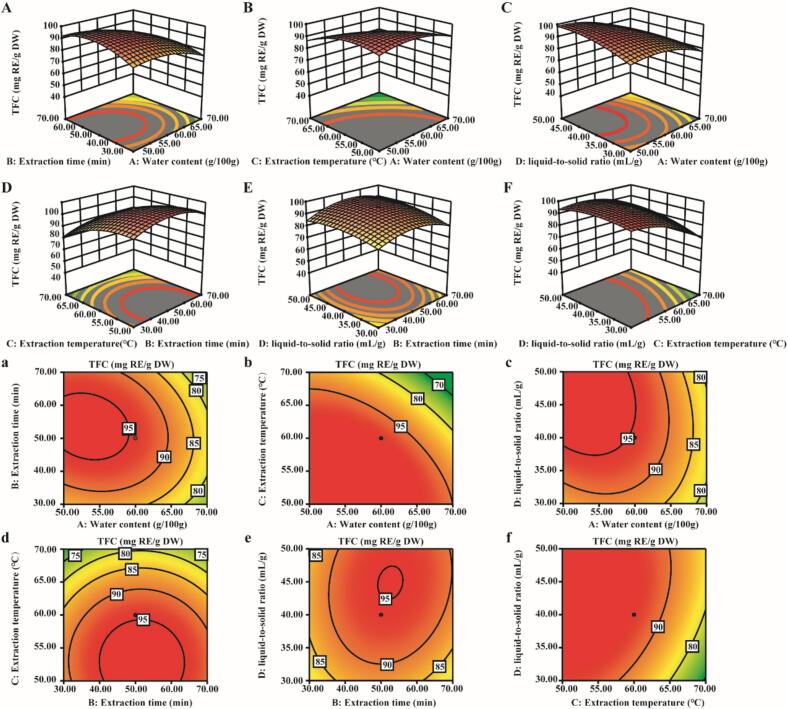
Response 3D surface plots (A-F) and contour plots (a-f) on TFC. Aa: water content and extraction time; Bb: water content and extraction temperature; Cc: water content and liquid-to-solid ratio; Dd: extraction time and extraction temperature; Ee: extraction time and liquid-to-solid ratio; and Ff: extraction temperature and liquid-to-solid ratio.

Fig. 2 presents the 3D response surface and contour plots of TFC extracted from AAF, illustrating the effects of four independent variables. These plots depict the interactive effects of variables on TFC response. Each graph shows the interaction effect of two independent variables on final TFC, with the other two variables held constant. Generally, an elliptical contour plot indicates a strong interaction between two variables, whereas a circular plot signifies little or no interaction [18], [52]). The TFC yield initially increased, reached a peak, and then slightly decreased with increasing water content (Fig. 2(A–C, a–c)) and extraction temperature (Fig. 2(B, D, F, b, d, f)) at lower levels, in alignment with the single-factor experiments. This behavior can be explained as follows: moderate water addition reduces NADES viscosity, thereby enhancing mass transfer and improving flavonoid diffusion. However, excessive water disrupts the hydrogen-bonding network of NADES, leading to reduced solvation capacity and lower extraction efficienc [42]. Similarly, increasing extraction temperature enhances diffusion kinetics by reducing solvent viscosity and improving mass transfer. However, at elevated temperatures, thermal degradation and structural decomposition of flavonoids become significant, leading to decreased yield, consistent with previous reports [59]. The liquid-to-solid ratio showed a positive effect on TFC extraction; however, beyond a certain threshold, no significant improvement was observed (Fig. 2C, E, F, c, e, f). Increasing solvent volume enhances the concentration gradient and improves contact between solvent and plant matrix, thereby promoting mass transfer. However, this effect becomes limited at higher solvent volumes due to system saturation, where further increases do not significantly enhance intracellular solute release [52].

As illustrated in Fig. 2(A, D, E, a, d, e), TFC initially increased, reached a maximum, and then slightly decreased with increasing extraction time. This trend is consistent with single-factor analysis. The initial increase is attributed to enhanced ultrasonic cavitation, which promotes cell wall disruption and accelerates flavonoid release. However, prolonged extraction may induce degradation of thermally and mechanically sensitive compounds due to continuous exposure to ultrasonic energy, temperature, and pressure fluctuations [18], [27].

According to the regression analysis, the optimized conditions for ChCl–LA-based NADES-UAE were determined as: 51.5 g/100 g water content, 56 min extraction time, 59 °C extraction temperature, and 48 mL/g liquid-to-solid ratio. Under these conditions, the predicted maximum TFC was 99.27 mg RE/g DW. 99.27 mg RE/g DW. The experimental value (98.71 ± 0.66 mg RE/g DW) closely matched the predicted value, with a relative error of only 0.56%, confirming the high accuracy, reliability, and predictive capability of the RSM model under NADES-UAE conditions.

### 3.3 Extraction kinetic study

To further characterize the NADES (ChCl-LA)-UAE process for extracting flavonoids from AAF and enable comparative evaluation, a kinetic study was conducted using ChCl-LA, 60% ethanol, and water as extraction media to support potential industrial applications. Extractions were performed under the optimized conditions described in Section 3.2.2, with extraction times ranging from 2 to 60 min.

**Fig. 3:**
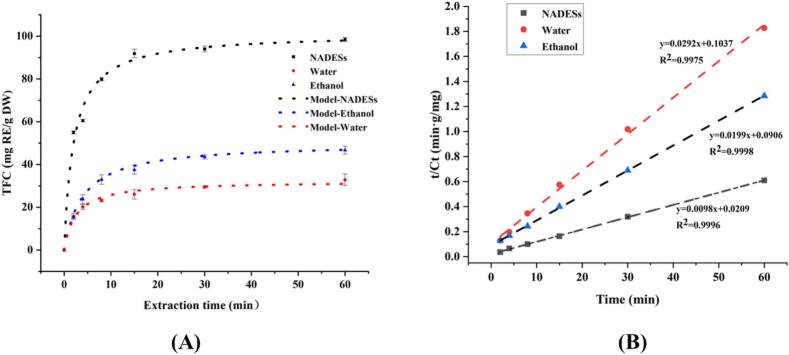
Kinetic curve of time extraction for Flavonoids from AAF.

As illustrated in Fig. 3, the ChCl–LA-based NADES significantly enhanced extraction efficiency, resulting in TFC values that were approximately 2–3 times higher than those obtained using ethanol or water. Notably, after only 2 min of extraction, the NADES system already exceeded the maximum TFC values achieved by conventional solvents, indicating a markedly accelerated extraction rate and improved solute availability. This behavior suggests that the NADES system substantially enhances the solubility and partitioning of flavonoids from the plant matrix, leading to rapid mass transfer at the initial stage. In the ChCl–LA-UAE system, TFC increased rapidly within the first 15 min, corresponding to the initial extraction phase. This stage is primarily governed by the synergistic effect of NADES-induced solubilization and ultrasound cavitation. The cavitation phenomenon generates microjets and shock waves that disrupt plant cell wall structures, increase membrane permeability, and facilitate rapid release of intracellular flavonoids, thereby significantly enhancing extraction efficiency. Similar enhancement mechanisms have been reported for DES-assisted ultrasound extraction systems, such as ChCl–malic acid, which also exhibit improved phenolic recovery from plant matrices [20]. In contrast, the second stage corresponds to a diffusion-controlled regime, where the extraction rate gradually decreases. This is attributed to the reduced concentration gradient as flavonoids migrate from the interior of plant particles to the solid–liquid interface. At this stage, mass transfer becomes governed by internal diffusion resistance rather than surface disruption, leading to a slower increase in extraction yield. This behavior is consistent with the “regular regime” described by Rakotondramasy-Rabesiaka [44], where extraction approaches equilibrium.

Overall, the kinetic results confirm that the ChCl–LA-based NADES-UAE system significantly enhances both initial extraction rate and overall mass transfer efficiency, compared with conventional solvent systems. These improvements are attributed to the combined effects of enhanced solvation capacity of NADES, and ultrasound-induced structural disruption of plant tissues, which together facilitate rapid release and diffusion of flavonoids from AAF.

### 3.4 FT-IR analysis

**Fig. 4:**
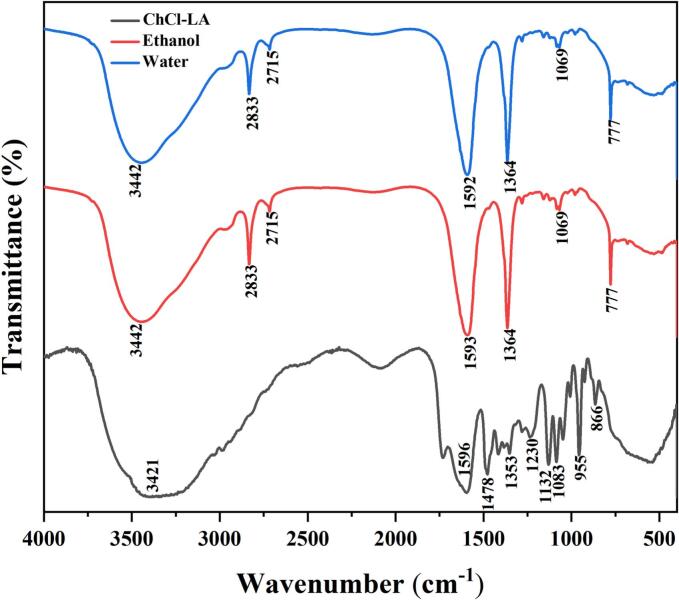
FT-IR of extracts from AAF using water, ethanol, and ChCl-LA.

In FT-IR spectroscopy, functional groups present within a molecule display absorption bands at specific characteristic frequencies. Fig. 4 presents the FT-IR spectra of the extracts, which were analyzed to investigate the structural modifications resulting from the sonication-enhanced synergistic effect of NADES (ChCl-LA), compared with those obtained using conventional solvents such as ethanol and water. The characteristic absorption band of flavonoids appears at 3442 cm^−1^ for conventional extractions, whereas the peak for flavonoids extracted with NADES is observed over a broader range (3500–3200 cm^−1^). Both signals correspond to inter- and intramolecular hydrogen-bonded O–H stretching vibrations of phenolic groups [36], [66]. This shift and broadening indicate that extraction using NADES promotes the formation of stronger and more extensive hydrogen-bonding interactions between flavonoids and solvent components, resulting in a modified vibrational environment of hydroxyl groups [65], [66]. The peaks observed at 2833 and 2715 cm^−1^ are attributed to C–H stretching vibrations, while those at 1596, 1593, and 1592 cm^−1^ correspond to aromatic C=C stretching vibrations within the flavonoid backbone [29], [36]. The bands at 1364 and 1353 cm^−1^ are associated with CH_3_ bending (scissoring) vibrations, whereas signals in the 1200–1300 cm^−1^ region are assigned to C=O stretching vibrations in phenolic and flavonoid structures. In addition, absorption bands in the 1050–1200 cm^−1^ region indicate C–O stretching vibrations, which are characteristic of flavonoid glycosidic and phenolic moieties [40]. Weak signals observed between 600 and 900 cm^−1^ are attributed to out-of-plane C–H bending vibrations of substituted aromatic rings, further confirming the presence of flavonoid structures [17].

Overall, the FT-IR spectra confirm the presence of flavonoids in both extraction systems. However, the NADES (ChCl–LA) extract exhibits stronger, more defined, and more complex absorption features, particularly in the fingerprint region (<1000 cm^−1^). This indicates that NADES-assisted extraction not only enhances flavonoid recovery but also preserves or stabilizes a wider range of functional groups, likely due to strong intermolecular interactions between NADES components and flavonoid molecules during extraction.

### 3.5 Scanning electron microscopy (SEM)

**Fig. 5:**
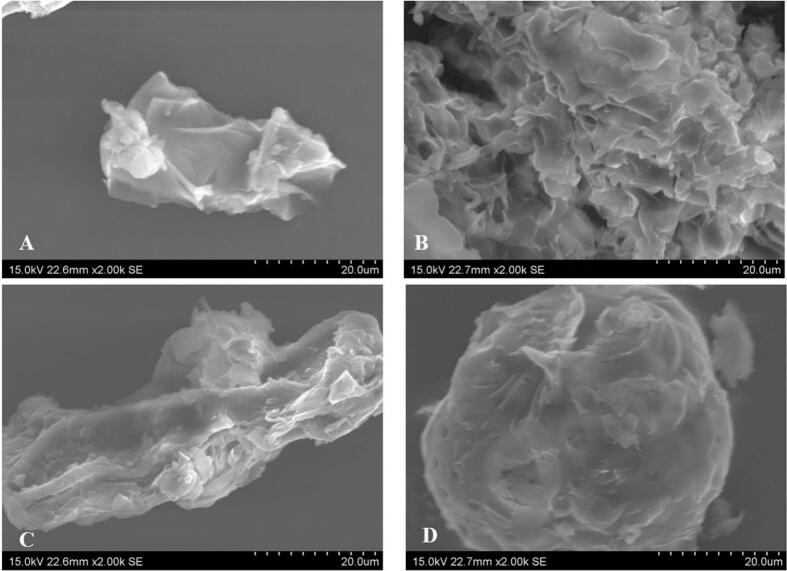
Effect of different extraction methods on SEM graphs: A, dried sample powder (AAFs); B, ChCl-LA; C, ethanol; D, water.

Ultrasonication combined with ChCl-LA significantly enhances mass transfer and accelerates the detachment of target analytes from the plant matrix [62]. The effectiveness of ultrasonic extraction is primarily attributed to the cavitation effect, a phenomenon driven by ultrasound-induced pressure oscillations characterized by alternating cycles of compression and rarefaction in the liquid medium [16], [61]. Therefore, the present study employed ultrasonication in combination with ChCl–LA to achieve synergistic extraction performance. As shown in Fig. 5(B), microscopic observations reveal pronounced structural disruption of plant tissues after NADES-UAE treatment, confirming the strong mechanical and physicochemical effects of the extraction system. In addition, the enhanced performance can be attributed to the high penetration ability and cell wall–disrupting capacity of ChCl–LA, as well as its strong affinity toward flavonoids in AAF. These combined effects result in more extensive exposure of intracellular structures, thereby facilitating solvent penetration and improving mass transfer efficiency [12]. In contrast, samples treated with conventional solvent systems (water and ethanol) exhibited relatively smooth and intact surface morphology (Fig. 5(C and D)), indicating limited cell wall disruption and weaker solvent penetration capability, which explains their lower extraction efficiency.

### 3.6 *In vitro* antioxidant assays

**Fig. 6:**
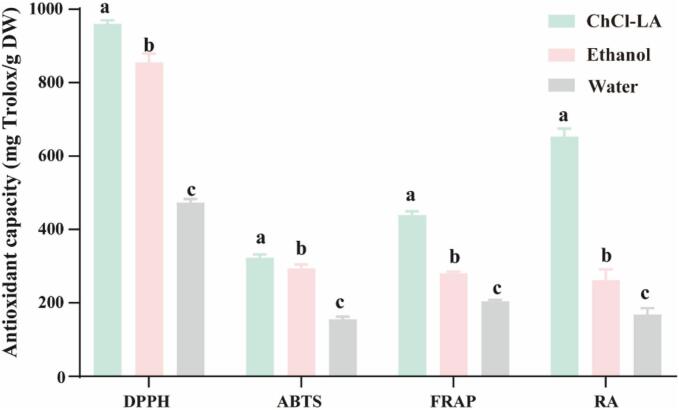
Antioxidant activities of AAF extracts using ChCl-LA, ethanol, and water.

To evaluate antioxidant potential, four in vitro assays—namely DPPH and ABTS radical scavenging, FRAP, and reducing activity (RA)—were employed to comprehensively assess the antioxidant capacity of AAF extracts. Fig. 6 shows that the extract obtained using ChCl-LA exhibited significantly higher values for all four antioxidant indices (DPPH: 959.98 ± 9.59 mg Trolox/g DW; ABTS: 322.97 ± 8.76 mg Trolox/g DW; FRAP: 280.51 ± 4.45 mg Trolox/g DW; RA: 652.68 ± 21.22 mg Trolox/g DW) compared to conventional solvent systems under optimized conditions as described in Section 3.2.2. Specifically, ethanol extracts showed DPPH: 854.88 ± 23.43 mg Trolox/g DW; ABTS: 294.33 ± 10.05 mg Trolox/g DW; FRAP: 439.18 ± 9.51 mg Trolox/g DW; RA: 262.14 ± 27.49 mg Trolox/g DW, while water extracts showed DPPH: 473.24 ± 9.16 mg Trolox/g DW; ABTS: 155.29 ± 7.20 mg Trolox/g DW; FRAP: 204.23 ± 3.61 mg Trolox/g DW; RA: 168.32 ± 16.35 mg Trolox/g DW. The results clearly demonstrate that ChCl–LA-based NADES-UAE extracts possess superior antioxidant activity compared with conventional extraction systems. This enhancement is primarily attributed to higher flavonoid yield and a broader spectrum of extracted phenolic compounds, which collectively contribute to improved radical scavenging and reducing capacity. In addition, this improved antioxidant performance suggests that the NADES-UAE system not only enhances extraction efficiency but also preserves the structural integrity and redox-active functional groups of flavonoids under mild extraction conditions, thereby maintaining their bioactivity. These findings are consistent with previous reports on NADES-assisted extraction systems, where improved antioxidant activity has been linked to enhanced recovery of phenolic compounds [19], [36].

The superior antioxidant activity observed in the ChCl-LA extract further supports the enhanced extraction efficiency and functional preservation achieved by the NADES-UAE system relative to conventional solvent extraction.

### 3.7 *In Vitro* Enzyme Inhibitory Activities of AAF extracts

With the increasing prevalence of diabetes, public health is increasingly threatened, posing a significant challenge for scientists. Starch, a primary dietary carbohydrate, when consumed in excess, can elevate blood sugar levels and disrupt glucose metabolism [25], thereby increasing the risk of diabetes [30], [54]. Consequently, controlling starch digestion through inhibition of key digestive enzymes, particularly α-amylase and α-glucosidase, is considered an effective strategy for managing postprandial hyperglycemia. Currently, acarbose is widely used as a clinical inhibitor of carbohydrate digestion. It slows starch breakdown, prolongs carbohydrate absorption, and reduces postprandial glucose spikes primarily through α-glucosidase inhibition [73]. However, long-term use of acarbose may cause adverse effects, including gastrointestinal discomfort and hypoglycemic episodes. Therefore, the development of natural enzyme inhibitors with fewer side effects has attracted increasing attention. Numerous studies have demonstrated that plant-derived extracts exhibit significant inhibitory activity against carbohydrate-hydrolyzing enzymes [27]. Flavonoids are recognized as key bioactive compounds responsible for such effects due to their ability to bind directly to α-glucosidase and form stable enzyme–inhibitor complexes. Their inhibitory activity is associated with hydrogen bonding, hydrophobic interactions, and modulation of the enzyme’s active-site microenvironment. In particular, hydroxyl groups on the flavonoid A-ring play a critical role in enzyme binding and inhibition [63]. Accordingly, targeting starch-hydrolyzing enzymes such as α-amylase and α-glucosidase represents a promising therapeutic strategy for natural antidiabetic agents [69].

**Fig. 7:**
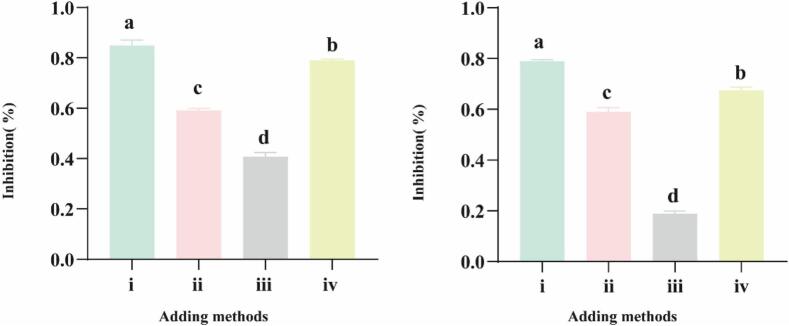
The effect of the addition method on the inhibition rates of A (α-amylase) and B (α-glucosidase). Data are presented as means ± SD (n = 5). Different lowercase letters (a, b, c, and d) above the bars indicate significant differences at *p* < 0.05.

In this study, four different sample addition methods were evaluated to determine the optimal interaction pathway between AAF extracts and enzyme systems (Fig. 7). Since flavonoids can interact with both starch and enzymes, the sequence of addition significantly influences inhibitory efficiency. Method I exhibited significantly stronger inhibition of both enzymes compared with other methods. This is attributed to extended interaction time between flavonoids and enzyme active sites, allowing more stable complex formation. Interestingly, Method IV showed higher inhibitory activity than Method II, which may be explained by non-covalent interactions between flavonoids and starch molecules, which can modify substrate availability and indirectly enhance inhibition [39]. Previous studies have confirmed that flavonoids can bind non-covalently to enzyme active sites via hydrogen bonding and hydrophobic interactions [55], [58]. These interactions collectively contribute to enzyme inhibition and delayed starch hydrolysis. Similarly, demonstrated that phenolic compounds are capable of binding to the amino acid residues of α-glucosidase through the formation of intermolecular hydrogen bonds involving hydroxyl groups located at the enzyme’s active site. These findings help explain how flavonoid compounds from AAF slow starch digestion not only through enzyme interactions but also via noncovalent binding with starch. Therefore, the first method (i) was identified as the most effective approach for maximizing the inhibitory activity of AAF extracts against α-amylase and α-glucosidase.

Furthermore, when comparing AAF extracts with acarbose (Fig. 7), at 125 μg/mL, AAF extracts inhibited α-amylase to a level comparable with 50 μg/mL acarbose, achieving approximately 84% inhibition. For α-glucosidase, AAF extracts achieved 91.8% inhibition at 125 μg/mL, which was slightly lower than 50 μg/mL acarbose (98.9%) but higher than 10 μg/mL acarbose (91.6%). These results indicate that the inhibitory performance of AAF extracts is enzyme-dependent and concentration-dependent, reflecting the multi-component nature of plant-derived bioactives. Unlike acarbose, which acts through a single-molecule mechanism, AAF extracts exhibit synergistic inhibitory effects arising from multiple flavonoid constituents, leading to broader but less targeted enzyme suppression.

These findings suggest that AAF flavonoid-rich extracts have strong potential as natural inhibitors of carbohydrate-hydrolyzing enzymes, particularly for α-glucosidase inhibition, and may serve as promising functional alternatives to synthetic antidiabetic agents.

### 3.8 *In Vitro* antibacterial activities of AAF extracts

The overuse and misuse of antibiotics and antimicrobial agents in recent decades have contributed to the emergence of resistant bacterial strains, including *Bacillus cereus*, *Staphylococcus aureus*, and *Escherichia coli*. In addition, antibiotic residues in the environment pose serious threats to human health by reducing treatment efficacy and promoting resistant infections [1], [9], [56]. Therefore, the development of safe, effective, and environmentally sustainable antimicrobial alternatives is of increasing importance.

Previous studies have reported that aqueous extracts of Artemisia argyi exhibit broad-spectrum antibacterial activity [71]. In this study, the antibacterial properties of AAF extracts were evaluated against three bacterial strains, including two Gram-positive bacteria (*S. aureus* and *B. subtilis*) and one Gram-negative bacterium (*E. coli*). The results obtained from agar diffusion assays are summarized in Table 4. The AAF extracts demonstrated antibacterial activity against all tested strains, with inhibition zone diameters of 12 mm for *E. coli*, 18 mm for S. aureus, and 29 mm for B. subtilis. Notably, the extracts exhibited the strongest inhibitory effect against *B. subtilis*. This difference in susceptibility may be attributed to variations in bacterial cell wall structure. In particular, Gram-negative bacteria possess an outer membrane rich in lipopolysaccharides, which acts as a protective barrier that limits the penetration of bioactive compounds, thereby reducing antibacterial susceptibility [3], [53].

**Table 4:** Antibacterial effects of AAF extracts on the bacteria.

**Type**	***E. coli***	***S. aureus***	***B. subtilis***
inhibition zone (mm)	12	18	29

Overall, these results confirm that AAF extracts possess broad-spectrum antibacterial activity, with higher efficacy against Gram-positive bacteria due to their relatively simpler cell wall structure, which allows easier diffusion of bioactive flavonoid compounds.

### 3.9 Application of AAF extracts in the preservation of strawberries

**Fig. 8:**
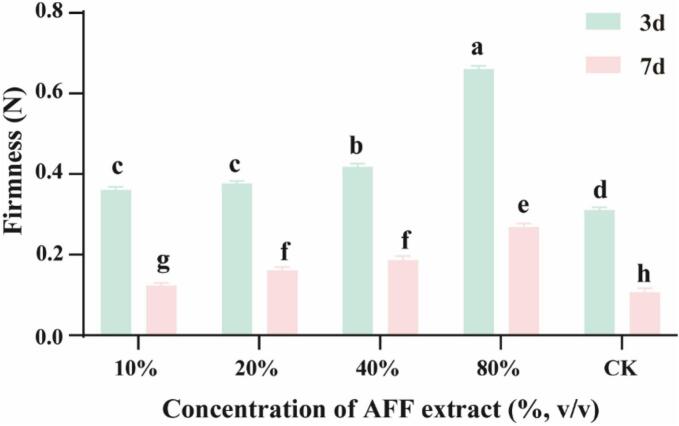
Changes in the firmness of strawberries treated with different concentrations of AAF extract. Data are presented as means ± SD (n = 3). Different lowercase letters within each sample indicate significant differences between treatments (*p* < 0.05).

The application of AAF extracts at different concentrations exerted a statistically significant effect (*p* < 0.05) on the firmness of strawberries. As illustrated in Fig. 8, the extract concentration played a crucial role in maintaining postharvest quality during storage. Even the lowest concentration of AAF extract resulted in a significant improvement in firmness compared with the control group throughout the storage period. Notably, firmness remained 15.9% higher than that of the control after 7 days of storage. In addition, strawberries treated with 80% AAF extract exhibited firmness values approximately 1.5-fold higher than those of the untreated control group.

Plants naturally activate both enzymatic and non-enzymatic antioxidant defense systems to respond to postharvest stress conditions [32]. In this study, the antioxidant defense response was evaluated by measuring peroxidase (POD) activity, a key enzyme involved in reactive oxygen species scavenging. In this study, the antioxidant defense response was evaluated by measuring peroxidase (POD) activity, a key enzyme involved in reactive oxygen species scavenging. POD activity was significantly higher in strawberries treated with AAF extracts compared with untreated controls. Specifically, strawberries treated with 80% AAF extract showed POD activities 1.02-fold and 1.15-fold higher than the control on days 3 and 7, respectively. This increase suggests that AAF extract may induce a mild stress response, thereby triggering the activation of endogenous antioxidant defense mechanisms. The presence of flavonoids in AAF extracts is primarily responsible for this effect, as these compounds are well known for their antioxidant and antimicrobial properties in plant-based preservation systems [32]. These flavonoids contribute to improved postharvest quality by enhancing membrane stability, reducing oxidative damage, and inhibiting lipid peroxidation processes [6], [26], [43], [51].

The findings demonstrate that AAF extract treatment effectively improves strawberry quality during storage. This improvement can be mainly attributed to the bioactive flavonoid fraction, which enhances antioxidant enzyme activity, reduces malondialdehyde accumulation, and preserves cellular structural integrity under oxidative stress conditions [38], [68].

## 4 Conclusion

This research successfully developed an effective and sustainable extraction system by integrating NADESs-UAE to valorize *Artemisia argyi* folium. The specifically formulated choline chloride-lactic acid NADES, coupled with UAE, demonstrated clear superiority over conventional aqueous and ethanolic extraction systems in flavonoid recovery, extraction kinetics, bioactivity, and preservation performance. Optimization of the extraction process established mild operational parameters. On the other hand, kinetic and microscopic investigations demonstrated that the extraction process is governed by diffusion, which is enhanced by efficient structural disruption of the plant matrix. Notably, the DES extract exhibited enhanced antioxidant and enzyme-inhibition activities compared with extracts obtained with ethanol or water. Additionally, the DES extract showed promising efficacy in preserving strawberries. The originality of this research lies in combining a customized, biocompatible DES with ultrasound technology to recover flavonoids from *Artemisia argyi* folium. Extensive mechanistic, kinetic, and phytochemical analyses substantiated the system's superior efficiency and selectivity. Furthermore, this foundational study proposes an effective, environmentally sustainable extraction model for bioactive compounds derived from underexploited plant resources. The approach provides a comprehensive framework for developing high-value natural products for the food, cosmetic, and pharmaceutical industries.

## CRediT authorship contribution statement

**Nina Bao:** Writing – original draft, Methodology, Funding acquisition, Formal analysis, Data curation, Conceptualization. **Xinyuan Zhao:** Investigation. **Lele Li:** Investigation. **Marwan M.A. Rashed:** Writing – review & editing. **Weifeng Yuan:** Investigation, Conceptualization.

## Declaration of competing interest

The authors declare that they have no known competing financial interests or personal relationships that could have appeared to influence the work reported in this paper.
